# Objective assessment of YAG laser vitreolysis in patients with symptomatic vitreous floaters

**DOI:** 10.1186/s40942-019-0205-8

**Published:** 2020-01-21

**Authors:** Carlos E. Souza, Luiz H. Lima, Heloísa Nascimento, Claudio Zett, Rubens Belfort

**Affiliations:** 1 Department of Ophthalmology, Universidade Federal de São Paulo, Rua Botucatu 821, Vila Clementino, São Paulo, SP 04023-062 Brazil; 2 Instituto Da Visão (IPEPO), São Paulo, Brazil; 3 Pontificia Universidad Católica de Valparaíso, Valparaíso, Chile

**Keywords:** Color fundus photograph, NEI VFQ-25 questionnaire, Vitreous floaters, Vitreolysis, YAG laser

## Abstract

**Background:**

To objectively evaluate YAG laser vitreolysis for symptomatic vitreous floaters using color photo imaging.

**Methods:**

In this interventional and prospective study, 32 eyes of 32 patients with symptomatic vitreous floaters secondary to posterior vitreous detachment (PVD) were treated with a single session of yttrium aluminum garnet (YAG) laser. Primary outcomes were objective and subjective changes measured by masked grading of color fundus photographs and National Eye Institute Visual Functioning Questionnaire 25 (NEI VFQ-25), respectively. Secondary outcomes included Early Treatment Diabetic Retinopathy Study (ETDRS), best-corrected visual acuity (BCVA) and adverse events. Wilcoxon signed-rank test was used to analyze the results of the objective and subjective assessments at each time point. P < 0.05 was considered statistically significant.

**Results:**

Thirty-two patients (32 eyes; 13 men and 19 women) with symptomatic vitreous floaters were enrolled in this study (mean age: 59.4 years). All study patients were followed up for 6 months. Following the laser vitreolysis, there was a statistically significant improvement in both the near visual function (z = − 2.97; p = 0.003; r = 0.633) and visual disturbance rate (z = − 3.97; p < 0.001; r = 0.84). Distance visual function did not show statistically significant difference after the laser procedure (p = 1.00). Color fundus photograph did reveal vitreous opacity improvement over time in 93.7% of study eyes (partial improvement in 37.5% and total improvement in 56.2% of study eyes). During the follow-up period, recurrence of vitreous floaters, BCVA deterioration and adverse events were not observed.

**Conclusions:**

YAG laser vitreolysis decreased the amount of vitreous floaters opacities seen on color fundus imaging and improved related symptoms according to the NEI VFQ-25 responses.

## Background

In the vitreous cavity, the hyaluronan maintains the fibrils of collagen separated, resulting in transparency of the vitreous gel. Nonetheless, vitreous liquefaction occurs as a part of the normal aging process and it is characterized by the dissociation between collagen and hyaluronan. This process leads to light scattering due to the collagen accretion with fibrous structures [1, 2]. Vitreous floaters represent a consequence of a posterior vitreous detachment (PVD) that enables the movement of vitreous body while the head or eye is moving, and it is clinically perceived as vitreous opacities that cause the appearance of dark spots in the center of vision [1, 2].

Vitreous floaters become more frequent in elderly people because of the degenerative vitreous changes that develop throughout life. They are common and usually harmless symptoms without any need for treatment [3]. The natural history of floaters is unpredictable with most of them resolving spontaneously and some worsening visual quality [3]. Although vitreous floaters represent a common symptom in patients, the majority of them accept the floaters perception without annoyance. Nonetheless, some patients such as pseudophakes and myopes may consider their symptoms disturbing [3, 4].

Patient education and observation, pars plana vitrectomy and laser vitreolysis represent the three management options for symptomatic vitreous floaters [5, 6]. With regards to laser vitreolysis, there are some reports on its efficacy to treat vitreous floaters. In these clinical studies, yttrium aluminium garnet (YAG) laser was applied in symptomatic patients to cause vitreolysis, and they showed good safety profile and improvement of symptomatic floaters estimated by the subjective criteria of questionnaire responses [7, 8]. The present study evaluated the efficacy and safety of YAG laser vitreolysis in a series of patients with symptomatic vitreous floaters, using the color fundus imaging objective assessment and the subjective information from National Eye Institute Visual Functioning Questionnaire 25 (NEI VFQ-25).

## Methods

In this double-center, interventional and prospective study, the floaters of study patients were treated with YAG laser vitreolysis at the Federal University of Sao Paulo (UNIFESP), São Paulo, Brazil, and Vision Institute, IPEPO, São Paulo, Brazil, from December 2017 to December 2018. The Institutional Review Board of the Federal University of São Paulo (reference number: 2.990.046) approved the off-label use of yttrium aluminium garnet (YAG) laser in the current study. All patients provided informed consent before treatment. The study was conducted in adherence to the tenets of the Declaration of Helsinki, and the methods and execution were consistent with the International Conference on Harmonisation Guidelines for Good Clinical Practice.

### Subjects

YAG laser vitreolysis was performed in 32 eyes of 32 consecutive patients (13 men and 19 women) with vitreous floaters due to PVD. All subjects had symptomatic vitreous floaters that were confirmed by biomicroscopy. None of the study participants had previously been treated for vitreous floaters.

Inclusion criteria were symptomatic floaters due to PVD, skill to undergo YAG laser vitreolysis, compliance of related risks, duration of floater symptoms of 6 months and beyond without evidence of regression, PVD demonstrated on both clinical examination and color fundus photographs. Exclusion criteria were best-corrected visual acuity (BCVA) worse than 20/70, history of glaucoma, retinal vein occlusion, diabetic retinopathy, retinal tear, retinal detachment, macular edema and uveitis.

The primary outcomes were objective and subjective changes measured by masked grading of color fundus photographs and self-reported percentage of vitreous floaters improvement and the near and distance activities subscale of the NEI VFQ-25, [9] respectively. Subjects answered the questionnaire, both before and after the procedure, and the improvements were based on the subjective responses. Secondary outcomes included Early Treatment Diabetic Retinopathy Study (ETDRS), best-corrected visual acuity (BCVA) and adverse events.

### Ophthalmologic examination, questionnaire evaluation and fundus photographs

Patients underwent a comprehensive ophthalmologic examination at baseline that included BCVA, biomicroscopic examination and intraocular pressure (IOP) measurement. All study patients were followed up for 6 months, and both ocular examination and color fundus photographs were performed at all follow-up periods (1-week, 2-week, 1-month and 6-month follow-ups). Color fundus photographs were performed with a Topcon TRC-50IA fundus camera (Tokyo Optical Co Ltd., Tokyo, Japan).

The subjective percentage of near and distance vision improvement following laser vitreolysis was analyzed using the NEI VFQ-25 questionnaire as described previously [10]. The patients were also asked to quantify the vitreous floaters improvement after laser vitreolysis based on a scale of self-rated visual disturbance, with 0 indicating no symptoms and 10 indicating debilitating symptoms.

The color fundus photographs were graded by one of us for the presence of floaters using a 5-level qualitative scale [7]. The following percentages were used to quantify the level of improvement: worse (less than 0%), same (0%), partial improvement (30% to 50%), significant improvement (50% to 70%), and complete improvement (100%).

### YAG vitreolysis

All laser treatments were performed by the same surgeon (CES). The study eyes were pre-operatively dilated with phenylephrine 2.5% and tropicamide 1%. Immediately before the YAG laser treatment, proparacaine was given, and a Volk Singh MidVitreous lens with goniosol was applied to the eye. The laser instrument used was the LIGHTLas YAG laser (LightMed, San Clemente, CA, USA) and it was properly aligned to an adequate crossing between the aiming beam and illumination beam. The YAG laser was initially set at its lowest energy of 0.5 mJ. Firstly, the laser beam was applied to single large floaters, breaking them into smaller opacities. Only one pulse per burst was performed and all participants underwent only one laser session. Post-operative topical medications were not prescribed. Laser vitreolysis was not performed if the vitreous floaters were located within 2 mm of the retina or the crystalline lens. The total number of laser shots performed per patient ended following the vaporization of the visually significant central fibrous opacities or Weiss ring.

### Statistical analysis

Data were analyzed using commercially available R-3.2.2 software. Wilcoxon signed-rank test was used to analyze the results of objective (grading of color fundus photographs) and subjective (NEI VFQ-25 questionnaire) assessments at each time point. We treated time as a categorical variable, and thus estimated means for the outcomes separately at each time point. P < 0.05 was considered statistically significant.

## Results

Thirty-two patients (32 eyes; 13 men and 19 women) with symptomatic vitreous floaters were enrolled. None of them had other ocular pathologies. The mean age of participants was 59.4 years (range 32–82 years). A PVD was observed in all subjects. The eyes that underwent YAG laser (62.5% phakic) received a mean of 366 laser shots and the mean average power per treatment session was 366.7 mJ (range 102–528 mJ. Both prepapillary (single membranous ring-shaped opacities or Weiss ring) and central (discrete and fibrous opacities floating freely around the centre of the vitreous cavity) vitreous opacities were treated with YAG laser. All 32 study patients were followed up for 6 months.

**Fig. 1:**
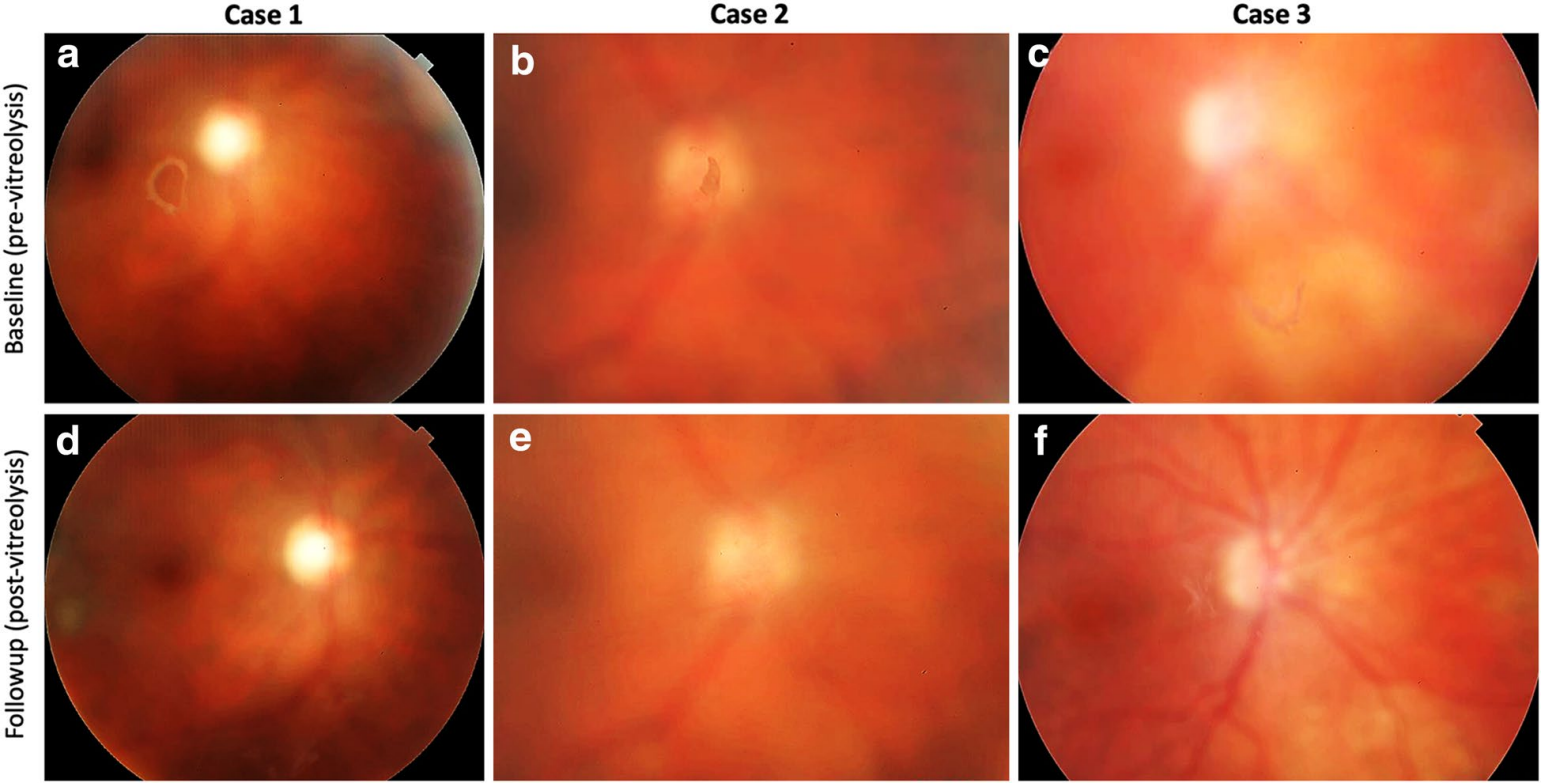
Color fundus photograph of three study cases at baseline before vitreolysis (**a**, **b** and **c**) and at 6-month follow-up (**d**, **e** and **f**). Note the vitreous opacity improvement over time

**Fig. 2:**
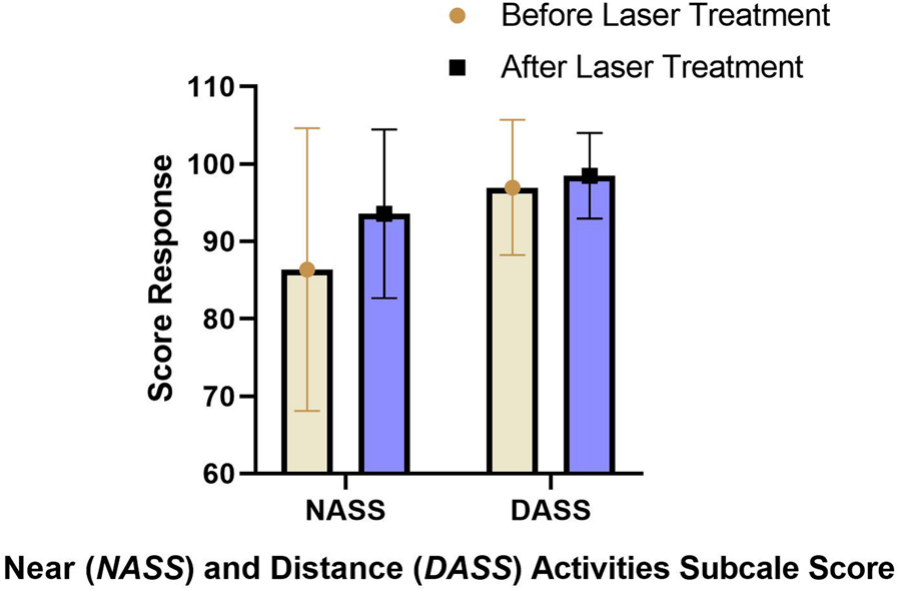
Before vs. after laser treatment subjective improvement using the NEI-VFQ near and distance activities subscale score (mean and standard deviation)

**Fig. 3:**
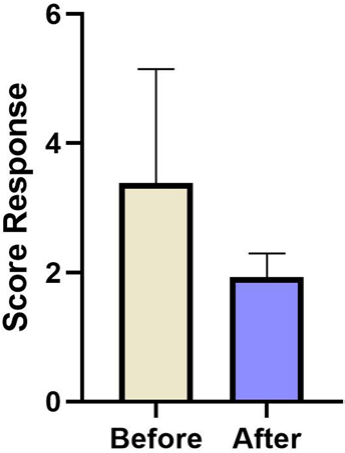
Subjective “visual disturbance rate” improvement before and after de laser procedure

Color fundus photograph revealed vitreous opacity improvement over time. A complete vitreous opacity improvement was observed in 18 eyes (56.2%), partial improvement in 12 eyes (37.5%), and no change in vitreous opacities in 2 eyes (6.3%) (Fig. 1). Following the laser vitreolysis, there was a statistically significant improvement in the near visual function (questions 5 to 7) compared with that before the procedure (z = − 2.97; p = 0.003; r = 0.633) (Fig. 2). In comparison with baseline, distance visual function (questions 8, 9 and 14) did not show statistically significant difference after the laser procedure (p = 1.00) (Fig. 2). Visual disturbance rate improvement showed a statistically significant reduction of 2.5 after the treatment (z = − 3.97; p = 7.62 < 0.001; r = 0.84) (Fig. 3). There was no change in the ETDRS BCVA during the 6-month follow-up period.

Increased intraocular pressure, intraocular lens dislocation, retinal tear, retinal detachment or recurrence of symptomatic vitreous floaters were not observed in the study patients during the follow-up period. In addition, none of the patients demonstrated significant visual decline or reappearance of vitreous floaters.

## Discussion

Currently, using optical coherence tomography (OCT) and contrast sensitivity, retinal specialists may detect the opacities their patients report and understand the reasons why vitreous floaters may affect the visual acuity of patients. Therefore, the management of vitreous floaters is becoming more proactive with the intention to treat floaters and improve patients’ quality of life. Novel treatment strategies supported by recent scientific studies and based on advances in YAG laser technology have become known to treat floaters (Table 1) [7, 8].

**Table 1:** Comparison between studies involving the use of Nd: Yag laser in the treatment of vitreous opacities

	No eyes	Mean power (mJ)	Improvement (%)^a^	Adverse events
Present study	32	366	46.1	None
Delaney et al.	39	1316	38.5	None
Shah et al.	52	310	53.0	One^b^

^a^ Improvement was based on answers of subjective questionaire. ^b^ One posterior chamber intraocular lens was pitted peripherally with the YAG laser when anterior floaters were treated

Although YAG laser is most commonly used for opacity of the posterior capsule following cataract surgery, application of YAG laser for the treatment of vitreous floaters has recently been reported. Several studies have demonstrated a great achievement rate related to laser vitreolysis without significant ocular complications [8, 11–13]. Using the objective analyses of color fundus photographs, the present study demonstrated vitreous opacity improvement following laser vitreolysis, and this improvement was observed in 93.7% of study eyes (partial improvement in 37.5% and total improvement in 56.2% of study eyes). The improvement of subjective outcomes corresponded to the improvement of floaters imaging on color fundus photographs analyses, and, therefore, supported the efficacy of laser vitreolysis. Statistically significant improvement in the near visual function was observed after a single laser session. However, distance visual function did not show significant difference after the single laser procedure.

In the present study, the patients stated that their floaters symptoms ameliorated in 46.1% following the YAG vitreolysis. Our findings appear to depict a slight difference regarding the improvement of floaters perception by the patients in comparison with those described in an analysis of 52 patients treated for symptomatic Weiss ring floaters from posterior vitreous detachment by Shah et al. [8]. In this report, a mean power of 1316 mJ per session was used, and no significant complication occurred during the 6 months of follow-up. 53% of patients reported significant or complete resolution of floaters symptoms. The difference in improvement of floaters perception between the current study (46.1%) and the study by Shah et al. [8] (53%) may be associated to the type of vitreous floaters that was addressed. In our study, both prepapillary Weiss ring and central discrete and fibrous vitreous opacities were treated with laser.

No adverse events judged to be of clinical relevance were observed after YAG laser vitreolysis in our sample. This result is corroborated by Singh et al. [12] that about 1300 eyes that he had treated with YAG laser vitreolysis. They looked specifically at adverse events and found that there was less than 1% risk of developing glaucoma, lens damage, or retinal damage. There were no cases of retinal detachment in this large series. Fifteen subjects voluntarily reported complications following YAG laser vitreolysis to the American Society of Retina Specialists Research and Safety in Therapeutics (ASRS ReST) Committee during a period of 6 months across the US [13]. Complications included elevated intraocular pressure leading to glaucoma, cataracts, including posterior capsule defects requiring cataract surgery, retinal tear, retinal detachment, retinal hemorrhages, scotomas, and an increased number of floaters. Our small sample was underpowered to identify less common potential complications and larger prospective studies will be critical to better understand the efficacy of the frequency, prognosis, and mechanism of associated complications.

Usually, visual acuity is determined by Snellen eye test and it is not significantly affected by floaters. The effects of floaters are better realized in other important aspects of visual acuity, such as contrast sensitivity and internal aberrations. Those aspects of visual acuity and how they relate to floaters were not widely understood in the past, and floaters were not recognized as a potentially significant visual complaint [1]. Color fundus photograph represents an imaging diagnostic technique that can determine just how much floaters affect a patient’s visual acuity and may have the potential to be used as an objective metric for vitreous floaters. Some reports have been tried to work toward finding some sort of objective metric to quantify and appreciate the degree of visual impairment that patients are experiencing [3, 6, 14]. Aberrometry can demonstrate the impact of coma that is induced by floaters, particularly when they move into the central visual axis. Usually the contrast sensitivity drops with floaters, especially if there is PVD [3]. In addition to contrast sensitivity and aberrometry, color fundus imaging could give further information that visual acuity does not. Color fundus imaging is able to show the amount of vitreous floaters that is impacting on patient´s symptoms.

## Conclusions

Color fundus imaging may have the potential to objectively assess the vitreous floaters changes following treatment with YAG laser vitreolysis in symptomatic patients. The present study does have several limitations, such as the lack of a control group, its small sample size and short follow-up period. Larger studies with longer follow-up periods are necessary to validate our described findings and to determine frequency of adverse events related to laser vitreolysis.

## Data Availability

We authorize the IJRV to reproduce any material described in the manuscript, including new software, databases and all relevant raw data.

## Abbreviations

BCVA: best-corrected visual acuity
ETDRS: early treatment diabetic retinopathy study
IOP: intraocular pressure
NEI VFQ-25: National Eye Institute Visual Functioning Questionnaire 25
OCT: optical coherence tomography
PVD: posterior vitreous detachment
UNIFESP: Federal University of São Paulo
YAG: yttrium aluminium garnet
